# Effects of Aspirin on Rheological Properties of Erythrocytes *In Vitro*

**Published:** 2012-09

**Authors:** Mohamed A. Elblbesy, Abdel Rahman M. Hereba, Mamdouh M. Shawki

**Affiliations:** *Medical Biophysics Department, Medical Research Institute, Alexandria University, Egypt*

**Keywords:** aspirin, erythrocytes, rheology

## Abstract

Aspirin is of proven value as an antithrombotic drug. In unstable angina it reduces the risk of death and myocardial infarction by half. Most studies on the mechanism of action of aspirin have concentrated on the effect of aspirin on platelets. In the present study we have tried to prove that there is another biophysical mechanism of aspirin, and that is through the effect of aspirin on erythrocytes. In this study ten blood samples were incubated with aspirin at different concentrations. The fractal dimension of erythrocytes subjected to shear rates from 5 s^-1^ to 30 s^-1^, in a cone and plate device designed and constructed in our lab, was calculated by processing the images of the erythrocyte. At each shear rate, the fractal dimensions of the erythrocytes were found to be strongly correlated with aspirin concentration. It is suggested that further studies using different biophysical methods must be carried out to detect the other mechanisms underlying the effect of aspirin on different blood cells.

## INTRODUCTION

Ingestion of aspirin (acetylsalicylic acid; ASA) reportedly affects many diverse physiological processes including histamine release, platelet aggregation, broncho-constriction, renal transplant rejection, and renal proteinuria. These effects are likely to be induced through the irreversible aspirin inhibition of the enzyme cycle-oxygenase, which is involved in the synthesis of prostaglandins and thromboxanes. Indomethacin is believed to work in a manner similar to aspirin, but its inhibition of the cycle-oxygenase is reversible. Salicylate is believed to bind reversibly to the cycle-oxygenase, but unlike aspirin or indomethacin, it does not inhibit the enzyme. Levels of estradiol and estrogen or other steroid hormones may modulate the effect of aspirin. Aspirin increases the bleeding time in men more so than in women, and women absorb more aspirin and clear aspirin more rapidly from the plasma than men. The incidence of ischemic heart disease among premenopausal women is much lower than among postmenopausal women and among men. Aspirin therapy has been implicated as an antithrombotic agent. Endogenous levels of estrogen are believed to maintain a more favorable ratio of prostacyclin to thromboxane which prevents platelet aggregation. Specific low dosages of aspirin enhance the prostacyclin to thromboxane ratio by inhibiting platelet thromboxane production with a smaller effect on vasculature prostacyclin production (1-3).

The beneficial cardiovascular effects of aspirin are generally attributed to its immediate platelet inhibitory function. However, accumulating evidence suggest that aspirin may have additional biological properties on the vasculature that contribute to the reduction of ischemic cardiovascular events in patients with hypertension and atherosclerosis (4).

It is well established that erythrocytes influence the interaction of platelets with vascular structures mainly through rheological mechanisms which may involve erythrocyte aggregation that should not be ignored. Rigidified erythrocytes have been demonstrated to cause an increase in platelet interaction with the subendothelium due to a more effective shift of the peripheral platelet and plasma layer towards the vessel wall. Hyperaggregation of erythrocytes is thought to play a similar role as it is associated with many cardiovascular disorders, but as yet there has been no experimental confirmation. To fully understand the role of erythrocytes in homeostasis and anti-thrombotic therapy, the application of few systems appears to be experimentally important. Methods employed in laboratory tests to assess the effect of drugs on blood cells are often performed in the absence of flow. These techniques provide results that are of limited value because rheological phenomena have not been taken into account. Perfusion methods allow this handicap to be overcome by simulating the physiological situation of the blood under flow (5, 6).

Aspirin has been intensively studied as a platelet anti-aggregant agent through irreversible inhibition of platelet cyclo-oxygenase. Furthermore, it has been reported that treatment of erythrocytes with ASA modifies platelet behavior. Dipyridamole (DIP) inhibits platelet aggregation in whole blood more than in isolated platelets, thus showing the relevance of erythrocytes in this process. It has been suggested that the anti-aggregating activity of DIP could be due to its inhibition of the active carrier-mediated uptake of adenosine by erythrocytes. DIP has been reported to increase erythrocyte deformability although its effect on erythrocyte aggregation is unknown. Association between ASA and DIP has been investigated in the laboratory, as well as in clinical trials, demonstrating synergism in the prevention of cardiovascular diseases (7).

In this study we have analyzed the effects of aspirin on erythrocytes at different shear rates and different aspirin concentrations, to quantify the influence on erythrocytes properties due to aspirin intake. Also this study tried to prove that the major effect of the aspirin is not only on the function of platelets but can also be extended to the other blood corpuscles, especially the erythrocytes, which constitute the major component of blood cells.

## MATERIALS AND METHODS

The local ethics committee of Medical Research Institute approved the initial research proposal, and all blood donors gave their informed consent of participation in the study. Ten healthy volunteers (male, age range 30-40 years) from a similar ethnic background were age- and sex-matched and had no history of clinical evidence of any systemic diseases, were included in this study. None of the blood donors had received any topical or systemic medication for at least 2 weeks before blood collection. None of our participants had a definite thrombotic risk.

### Sample Preparation

Five ml of blood was collected from each donor and EDTA was used as anticoagulant. The whole blood sample was centrifuged at 3000 rpm to separate erythrocytes. Then, Erythrocytes were washed twice in phosphate buffer saline (PBS). Aspirin solutions with concentrations of 0.01 mg/l, 0.02 mg/l, 0.03 mg/l, 0.04 mg/l and 0.05 mg/l were prepared by dissolving of aspirin tablets in PBS. Erythrocytes were suspended in aspirin solution by adding 1 ml erythrocytes to 1 ml of aspirin solution. After one hour erythrocytes were centrifuged and washed three times in PBS in order to remove aspirin solution.

### Fractal analysis of erythrocytes

The aggregation and dynamic rheology of erythrocytes were studied using shearing chamber with cone-plate geometry. The shearing chamber hada radius 20 mm and angle 5°. The shearing chamber was connected with stepper motor in order to change shear rate. Our shearing chamber was designed to give shear rate range from 5 s^-1^ to 50 s^-1^.

Erythrocytes treated with aspirin were resuspended in PBS at 5% concentration and 2 μl of erythrocytes suspension was introduced in shearing chamber. The shearing chamber was built on the stage of inverted microscope which is coupled with CCD camera. CCD camera was connected to computer through an interface. Erythrocytes suspension was shearing at 5 s^-1^, 10 s^-1^, 20 s^-1^, and 30 s^-1^ for 2 minutes. For each shear rate value ten shots of erythrocytes images were taken and transferred to computer. The erythrocytes aggregation was computed by calculation of erythrocytes fractal dimension. Images of erythrocytes under different shear rate were transferred to computer in order to make image processing for each image using HarFA free software. Fractal dimension for each image was determined directly through order fractal dimension in the program tool bar (8).

### Adhesion Number

Erythrocytes treated with aspirin were used to prepare erythrocytes suspension in PBS of the following volume fraction (ϕ): 0.005, 0.01, 0.015, 0.02, 0.025%. 20 µl of erythrocytes were placed on incline glass plate and left for 2 min in order to reach steady state. The glass plate was then observed by microscope which had been coupled to computer through CCD camera and interface. For each plate ten shots were taken. The number of doubles erythrocytes (N_2_) and single erythrocytes (N_1_) was counted for each plate in order to calculate the adhesion number. The adhesion number was calculated as the slope of the straight line of the relation between (N_2_/N_1_) and the volume fraction (ϕ) of the erythrocytes (9).

### ErythrocytesDeformability

Erythrocytes deformation index (DI) was used as indicator for erythrocytes deformation. DI was calculated by image analysis of erythrocytes images under different shear rates for control and erythrocytes treated by aspirin. Images were digitized and analyzed using image processing software. Cells were automatically located in each image and the contour of each cell projection was fitted by an ellipse, allowing estimating the major (*a*) and minor (*b*) axes of the cell as well as the orientation angle with respect to the streamlines of the flow. DI was calculated as the following:

(i) $$DI\;=\;\frac{a}{b}$$

### Osmotic Fragility

The osmotic fragility of RBCs was determined by using series of NaCl concentrations; ranged from 0% (distilled water) until normal saline (0.9% NaCl). The test was carried out within 1 hour of collection. 20 μL RBCs was added to 2 mL of each NaCl concentration with gentle mix. The samples were incubated for 30 minutes in the dark, then centrifuged at 2000 rpm for 15 minutes (10). The hemolysis percentage (% H) was calculated from the following equation:

(ii) $$\%\;\text{H}\;\text{at}\;\text{certain}\;\text{concentration}\;=\;\frac{\text{A}\;\text{at}\;\text{this}\;\text{concentration}}{\text{A}\;\text{at}\;0\%\;\text{concentration}}\;\times \;100$$

where A is the supernatant absorption using spectrophotometer (Shimadzu UV model 1601 Spectrophotometer) at 540 nm.

### Hemoglobin Spectrum

20 μL of RBCs was mixed with 2 mL distilled water to ensure complete hemolysis. The samples were incubated for 30 minutes in the dark, and then centrifuged at 2000 rpm for 15 minutes. The absorbance intensity of the hemoglobin solution for each sample was measured using UV/visible spectrophotometer (JENWAY, 6305, USA) at wavelength range from 320-600 nm (11).

## RESULTS

**Figure 1:**
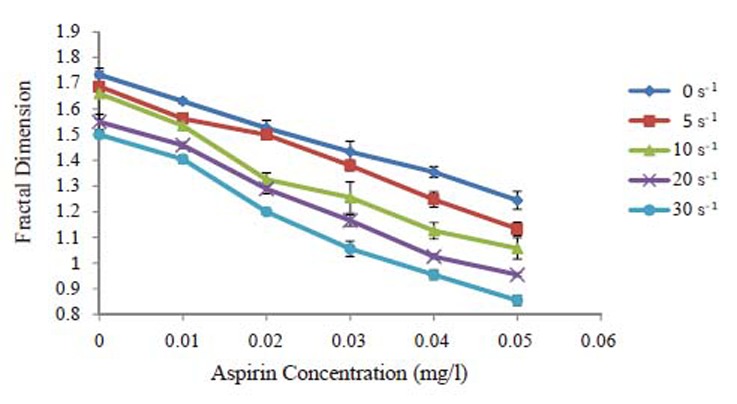
The relation between fractal dimension and aspirin concentration under different shear rates.

The effect of aspirin concentration on erythrocytes fractal dimension is given in Fig. 1. The fractal dimension values decrease as the aspirin concentrations increase. Also it is clear that as the value of shear rate increases the fractal dimension of erythrocytes decreases. There is a strong correlation between fractal dimension of erythrocytes and aspirin concentration for all values of shear rates. In comparison between the values of fractal dimension at zero shear rate and higher values of shear rate it is clear that the fractal dimension at 0 s^-1^ is higher than the other values of shear rates for all erythrocyte suspended in different values of aspirin concentration. Control samples which are untreated with aspirin give higher values of fractal dimension than the treated ones.

**Figure 2:**
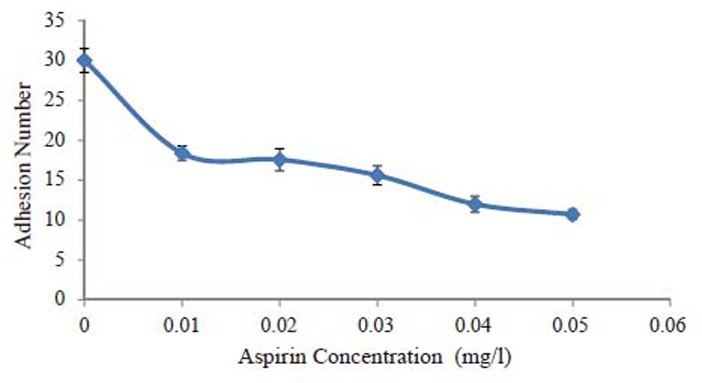
The relation between adhesion number of erythrocytes and aspirin concentrations.

Fig. 2 shows the relation between the adhesion number of erythrocytes and aspirin concentration. It is clear that the adhesion number of control is greater than that of erythrocytes exposed to aspirin. The value of adhesion number is reduced by approximately 40% when erythrocytes were exposed to aspirin with 0.01 mg/l and by 64.4% when they were exposed to aspirin with concentration of 0.05 mg/l. The decrease of adhesion number at the beginning is dramatically large but the difference among the adhesion numbers of the erythrocytes exposed to aspirin with different concentrations is small.

**Figure 3:**
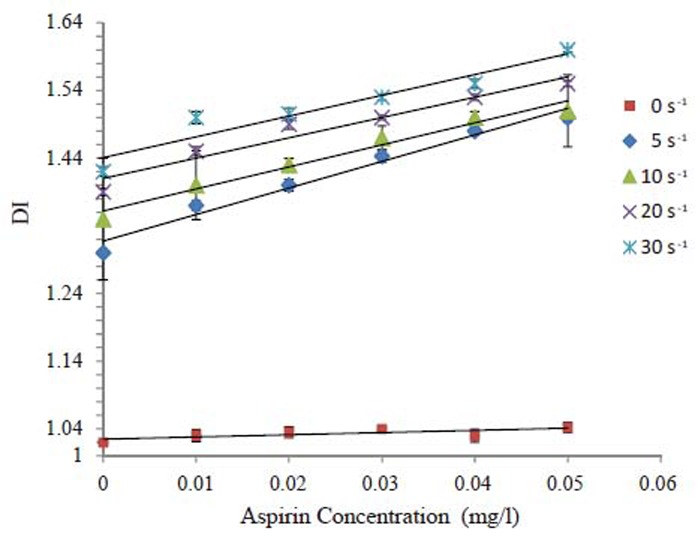
Deformation of erythrocytes due to different concentrations of aspirin and different values of shear rates.

DI values were increased due to treatment of erythrocytes with aspirin. The increase in DI values for erythrocytes is very clear in comparison with control. From Fig. 3 the relation between aspirin concentrations and DI is directly proportional. Also, the aspirin increases the deformability of erythrocytes by about 76 % at 0 s^-1^. The combination effect of aspirin and shear rates increased the deformability of erythrocytes. A strong correlation (R^2^>0.5) was observed for the relation between DI and aspirin concentrations under different values of shear rates.

**Figure 4:**
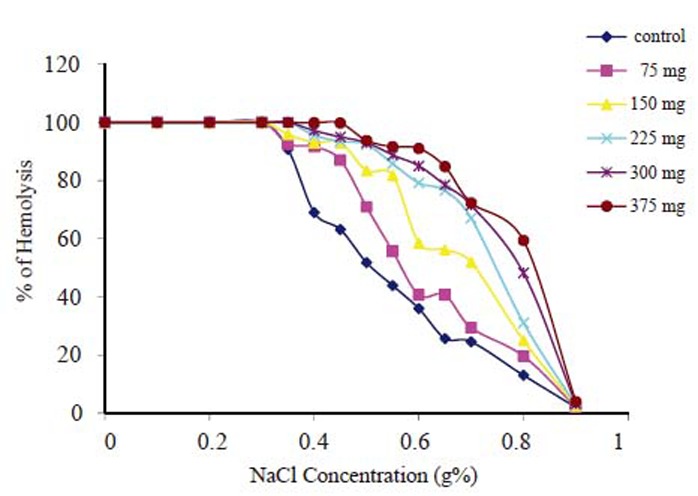
Percentage of erythrocytes hemolysis at different aspirin concentrations.

Fig. 4 shows the results of osmotic fragility measurements for control sample and all aspirin incubated samples, where the percentage of hemolysed cells is plotted as a function of the concentration percentage of NaCl. From Fig. 4 it is possible to calculate the median corpuscular fragility (MCF); (the NaCl concentration at which 50% of RBCs are hemolyzed) for each group. It was found that there is significant increase (*p*<0.05) of MCF as the aspirin concentration increasesas shown in Table 1.

**Table 1:** The median corpuscular fragility (MCF) for each group

Group	Control	75 mg	150 mg	225 mg	300 mg	375 mg

MCF (g%)	0.49 ± 0.03	0.57 ± 0.02	0.75 ± 0.01	0.77 ± 0.01	0.81 ± 0.02	0.85 ± 0.04

**Figure 5:**
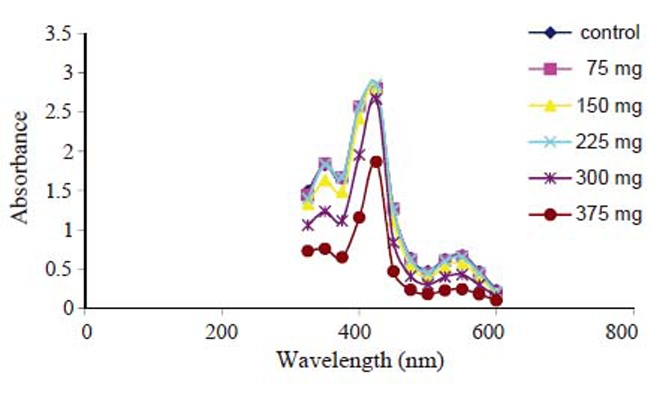
Hemoglobin absorption spectra.

Hemoglobin absorption spectra (Fig. 5) indicate the appearance of the well known hemoglobin characteristic bands at 340, 410, 540, and 578 nm. These bands correspond to globin-heme interaction, soret band, nitrogen ion bonds in porphyrine rings and heme-heme interaction bands respectively. There is significant decrease in these bands only with 300 mg and 375 mg aspirin groups compared to the control.

## DISCUSSION

Clinical and laboratory research provide clear evidence that the platelets activation is partly induced by stimuli from other kinds of blood elements (12, 13). The beneficial cardiovascular effects of aspirin are generally attributed to its immediate platelet inhibitory function. However, accumulating evidence suggest that aspirin may have additional biological properties on the vasculature that contributes to the reduction of ischemic cardiovascular events in patients with hypertension and atherosclerosis (14, 15). Yousif Y. Bilto suggested that aspirin could play a rheologically active role on erythrocytes and stated that aspirin could be explained by acetylation of intracellular proteins and hence saturation (concentration) of the cell interior with the osmotically active drug (16). The results obtained by A. El Bouhmadi *et al* confirmed that erythrocytes aggregation is increased under oral contraceptives and 100 mg aspirin acutely can induce a partial reversal of this erythrocytes hyper-aggregation (17). Our study shows that the status of erythrocytes aggregation, indicated by erythrocytes fractal dimension, is affected by aspirin concentration which indicates that the effect of aspirin on platelets can be mediated through the effect of aspirin on other blood components especially erythrocytes. In this study; it is shown that as the concentration of aspirin increases the fractal dimension decreases hence the erythrocytes aggregation decreases. Gergely Fehe *et al* had stated that patients with effective acetylsalicylic acid inhibition had significantly lower plasma fibrinogen level and red blood cell aggregation values (18). This seems to be in accordance with our results, but in our study the erythrocytes were suspended in PBS not plasma and this indicates that aspirin has a significant effect not only on plasma proteins but also on the erythrocytes.

Robert S. Rosenson showed that aspirin/dipyridamole was more effective than aspirin therapy in reducing blood viscosity at shear rates of 1 s^-1^ and 2 s^-1^; however, there were no significant differences in blood viscosity at shear rates of 5 s^-1^ to 1000 s^-1^ (19). As shown in Fig. 1 as the shear rates increase the fractal dimensions decrease, this result indicates that there is also an effect of the dynamic properties of the blood combined with the effect of aspirin.

The osmotic fragility of RBCs, which reflects the membrane’s ability to maintain structural integrity is shown in Fig. 4. The results indicate that as aspirin concentration increases, the hemolysis percentage increases which is in agreement with many previous studies which state that at the biochemical level aspirin alters red cell membrane functions by inhibiting cholinesterase, ion-dependent ATPase activity and by altering the ion permeability across the cell wall (20-22). With these defects along with altered cell shape, it is reasonable to assume that the cells may be more susceptible to intravascular hemolysis. Hemoglobin pigment was detected in some of the renal elements and free hemoglobin was also detected in the plasma of some of the acutely treated mice (23). Both these observations are reported as indicators of hemolysis *in vivo* (24).

(Durak *et al.*, 2001) stated that aspirin even at normal dose (10 mg/kg/day) can cause peroxidation in human erythrocytes, increasing glutathione peroxidase and catalase activities but without changing the susceptibility to oxidation (25). This increase in free radicals can interfere with hemoglobin molecule causing partial structural changes in the Hb molecules which was found in hemoglobin spectrum as shown in Fig. 5.

Lanas *et al* had reported that 30% of patients with a history of aspirin related gastrointestinal bleeding have an exaggerated prolongation of skin bleeding time in response to aspirin, which may be a risk factor for bleeding (26). Lucia Mannini *et al* concluded that in patients with acute coronary syndromes the antiaggregant effect of aspirin is modulated not only by the direct action on platelets, but also by erythrocyte deformability and white blood cells count (27). Teresa Santos *et al* suggested that low-dose antithrombotic therapy with aspirin can be rendered more efficient and the prothrombotic effect of erythrocytes neutralized (28). Our findings in this study show that there is a clear effect of aspirin on the energy of adhesion between erythrocytes which forms another side of the effect of the aspirin on the erythrocytes which must be taken in consideration when we study the biophysical mechanisms of aspirin.

## CONCLUSION

From this study we conclude that there are other biophysical mechanisms, beside the role of aspirin in platelets function, which must be taken into consideration. Further study using different biophysical methods must be carried out in order to clarify the effect of aspirin not only on platelets but also on the other blood components.
